# Safety and Efficacy of Intracavernosal Injections of AbobotulinumtoxinA (Dysport^®^) as Add on Therapy to Phosphosdiesterase Type 5 Inhibitors or Prostaglandin E1 for Erectile Dysfunction—Case Studies

**DOI:** 10.3390/toxins11050283

**Published:** 2019-05-21

**Authors:** Francois Giuliano, Charles Joussain, Pierre Denys

**Affiliations:** 1 Neuro-Uro-Andrology R.Poincare academic hospital, AP-HP, 104 bvd R. Poincare, 92380 Garches, France; charles.joussain@uvsq.fr (C.J.); pierre.denys@aphp.fr (P.D.); 2 Faculty of Medicine, Versailles Saint Quentin University, Paris Saclay, 78180 Montigny le Bretonneux, France

**Keywords:** botulinum toxin A, combination therapy, non-responders, penile erection, pharmacology, sympathetic overactivity, pathophysiology, mechanism of action

## Abstract

Erectile dysfunction (ED) is a highly prevalent condition with a variety of possible risk factors and/or etiologies. Despite significant advances regarding ED pharmacological management, there are still insufficient responders to existing pharmacological treatments e.g., approximately 30% of patients are insufficient responders to phosphodiesterase type 5 inhibitors (PDE5-Is). It has been recently proposed that botulinum toxin A intracavernosally (IC) delivered could be effective in these patients. Data from a retrospective uncontrolled single center study of 47 ED patients, consecutively recruited, insufficient responders to existing pharmacological treatments e.g., PDE5-Is or IC PGE1 injections treated with IC abobotulinumtoxinA 250 or 500 U as free combination with their existing treatment have been analyzed. Response rate, according to the International Index of Erectile Function-Erectile Function domain score, 6 weeks following IC abobotulinumtoxinA in combination with prior pharmacological treatment, was 54%. Two patients have reported mild penile pain on injection or during the 3 days following injection. Therapeutic efficacy did not seem to be influenced by the etiologies and/or risk factors for ED. Conversely, the less severe ED, the higher the response rate. Preliminary evidence for the therapeutical potential with acceptable safety of IC abobotulinumtoxinA as add-on therapy for ED not sufficiently responsive to standard therapy should be confirmed in randomized clinical trials.

## 1. Introduction

Erectile dysfunction (ED) is not a disease but a symptom. ED is defined as the inability to achieve or maintain an erection sufficient for satisfactory sexual performance [1]. Penile erection is a complex phenomenon which implies a delicate and co-ordinated equilibrium among the neurological, vascular and the tissue compartments. It includes arterial dilation, trabecular smooth muscle relaxation, and activation of the corporeal veno-occlusive mechanism [2]. The pathophysiology of ED may be vasculogenic, neurogenic, psychogenic, and/or drug-induced [2]. ED prevalence varies from one region of the globe to another. Below the age of 40, prevalence of ED is 1–10%. In the fifth decade, the prevalence ranges from 2% to 9%, as high as 15%. The 50–59 years of age group shows the greatest range of reported prevalence rates. Most of the world showed a rather high rate of 20–40% for the ages 60–69 years. Almost all the reports showed a higher prevalence rate for those men in their 70s and 80s ranging from 50% to 100% prevalence in these decades [3]. ED risk factors include age, smoking, diabetes mellitus, hypertension, dyslipidemia, metabolic syndrome, obesity and sedentary lifestyle [4]. Compelling evidence exists showing that the most common underlying mechanism of ED is vascular and that cardiovascular diseases and ED share etiologies as well as pathophysiology [5]. Chronic neurologic conditions including spinal cord injury, multiple sclerosis and stroke are often associated with ED. ED is also common after surgery or radiotherapy for prostate cancer. The association of ED with depression is also well established. In addition, many antidepressants can lead to ED [3,4].

Medical treatment of ED was revolutionized by the advent of phosphodiesterase type 5 inhibitors (PDE5-Is) 21 years ago. There is robust evidence that PDE5-Is are effective, safe and well-tolerated therapies for the treatment of ED. Whatever the etiology of ED, PDE5-Is are first line therapy for most men with ED who do not have a specific contraindication to their use [6]. PDE5-Is targets the nitric oxide (NO)-cGMP relaxing pathway within erectile tissue. Sexual stimulation leads to the release of NO by cavernosal nerve endings and endothelial cells lining the sinusoidal spaces of the corpora cavernosa. NO diffuses into cavernosal smooth muscle cells to stimulate guanylate cyclase and generate cGMP production. Hence, there is an activation of cGMP-dependent protein kinase G which in turn phosphorylates several proteins. These protein kinase interactions result in reduced intracellular calcium levels and a consequent relaxation of arterial and trabecular smooth muscle, leading to arterial dilation, venous constriction, and the rigidity of penile erection [7]. Lack of proper cGMP elevation may result from insufficient release of NO from nerve endings and/or endothelial cells. PDE5-Is enhances erectile response to sexual stimulation by inhibiting PDE5 which is the enzyme that degrades cGMP. The decrease in cGMP degradation maintains higher levels of cGMP thereby increasing the relaxation of vascular and cavernosal smooth cells. Thus, there is an increase in blood flow to the penis and an increased filling of sinusoidal spaces restoring normal erection [6]. Despite global effectiveness of PDE5-Is in ED with psychogenic and/or organic causes, it has been estimated that overall there are 25 to 35% non-responders to PDE5-Is [8,9,10]. Local pharmacological therapies represent second-line therapy and should be considered in patients in whom PDE5-Is are not effective [6]. Prostaglandin E1 (PGE1) or alprostadil has been approved worldwide for intracavernosal injections (ICI) therapy. When injected within the erectile tissue of the corpora cavernosa, subjects are capable of initiating and maintaining erection without any sexual stimulation.

Thirty years ago, it was already suggested that botulinum toxin could become “a local aphrodisiac” as “a carefully measured locally injected dose should subtly weaken the contractile power of the penile arterioles. For afterwards, erection should be much easier to achieve and maintain; and the treatment can be easily repeated” [11].

One randomized controlled trial has been conducted involving 24 patients with severe vasculogenic ED refractory to PDE5-Is and to ICI of tri-mix (a custom-prepared combination of three drugs: alprostadil or PGE1, papaverine and phentolamine). These patients were candidates to penile implant surgery [12]. The treated group received a single ICI of onabotulinumtoxinA (Botox^®^) 50 U while the control group received an injection of 1 mL of saline 0.9%. In the treated group, an improvement in penile vascular parameters assessed by penile color duplex was reported 2 weeks after treatment and an improvement in the Sexual Health Inventory for Men (SHIM) score [13] and in the Erection Hardness Score [14] 4 weeks after treatment. With on-demand sildenafil 100 mg, 3 out of 12 patients from the treated group were able to achieve an erection lasting long enough to complete sexual intercourse versus none in the control group 4 weeks after treatment. One patient in the treatment group experienced a prolonged erection following IC tri-mix injection performed at the time of penile color duplex. There was no episode of priapism nor systemic toxicity [12]. While encouraging, these results pointed out that the efficacy could likely be improved by increasing the dosing of onabotulinumtoxinA. The same group of investigators has accordingly managed to further study the effect of IC delivery of onabotulinumtoxinA 100 U in two separate clinical trials in patients with vasculogenic ED (ClinicalTrials.gov Identifier: NCT03355963 and NCT03102762).

Three preparations of botulinum toxin A (BTX-A) are commercially available and approved by the United States Food and Drug Administration i.e., onabotulinumtoxinA, abobotulinumtoxinA and incobotulinumtoxinA. None of which are identical or interchangeable. Dysport^®^/Reloxin^®^/Azzalure^®^ are the trade names for abobotulinumtoxinA. It was approved by the FDA in 2009 for the treatment of cervical dystonia and for temporary improvement in the appearance to moderate-to-severe glabellar lines. The difference between Botox^®^ (onabotulinumtoxinA) and Dysport^®^ (abobotulinumtoxinA) lies in the purification procedure. Botox^®^ is purified by repeated precipitation and re-dissolution, whereas Dysport^®^ is purified by a column separation method [15]. Dose ratios between Dysport^®^ and Botox^®^ have been debated in the past and likely vary greatly based on dosage [15].

In order to further investigate the safety and efficacy of BTX-A for the treatment of ED, we report retrospective data from an open label study of intracavernosal abobotulinumtoxinA (Dysport^®^) as add on therapy to PDE5-Is or PGE1 ICI for the treatment of adult men with ED to improve erectile function when this has not been provided by PDE5-Is or PGE1 ICI alone.

## 2. Results

### 2.1. Patients Demographics

The mean age of the 47 patients was 55.1 ± 9.7 (min: 28, max: 82). Average duration of ED was 8.4 ± 6.6 years. Etiology(ies) and/or risk factor(s) for ED are summarized in Table 1.

**Figure 1:**
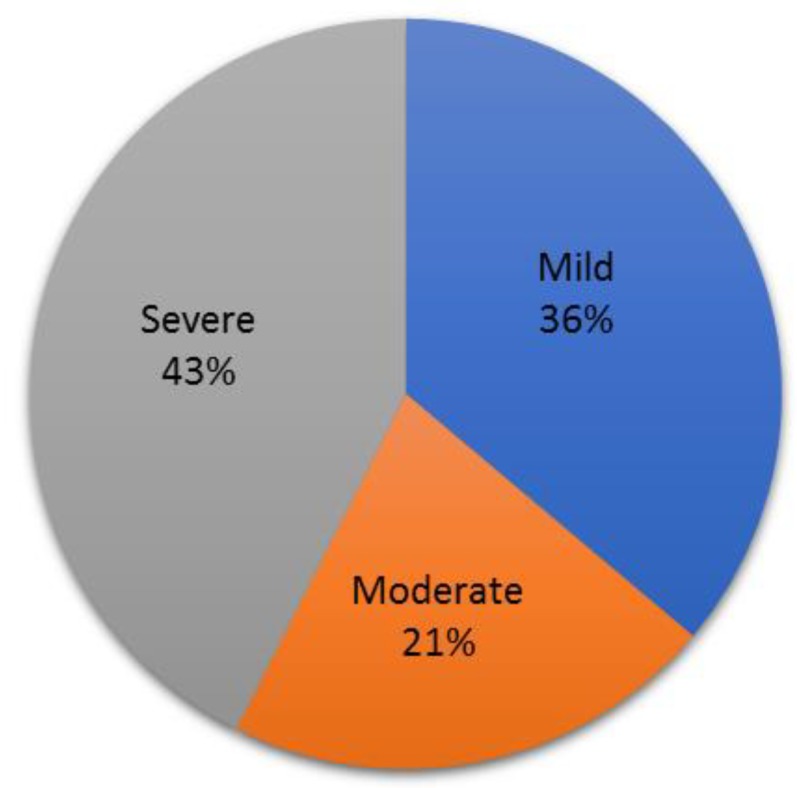
Classification of 47 patients according to the degree of severity of ED under existing pharmacological treatments at baseline prior to IC abobotulinumtoxinA based on the EF-IIEF domain score [16].

Thirty-three and 20 patients have been treated by IC abobotulinumtoxinA 250 and 500 U respectively. Among the 20 patients treated with abobotulinumtoxinA 500 U, 12 were BTX-A IC naïve, 3 were not responders to abobotulinumtoxinA 250 U, and 5 were responders to a lower dose of BTX-A IC and requested second injections because of the decrease over time of the efficacy of the first injections. Pharmacological ED treatment(s) providing an insufficient response at baseline were PDE-Is highest approved dose on demand: Either 100 mg sildenafil, 20 mg vardenafil, 20 mg tadalafil, or 5 mg daily tadalafil in 35 patients (74.5%) or PGE1 ICI (average dose 36.4 ± 15.5 µg) in 14 patients (29.8%). Two patients were treated by PDE5-Is combined with PGE1 ICI. Under treatment, IIEF-EF domain score prior to abobotulinumtoxinA IC injections was 13.1 ± 6.1. Patients were categorized according to severity of ED under treatment at baseline prior to abobotulinumtoxinA IC injections based on their EF-IIEF domain score [16] (Figure 1). Among the 47 treated patients with abobotulinumtoxinA, one was lost of follow-up prior to the first follow-up visit. Thus, safety has been assessed in 46 patients, while in 2 out of these 46 patients, efficacy was not evaluable.

### 2.2. Safety

One patient spontaneously reported mild penile pain on IC injection. Another one reported mild penile pain during the 3 days following IC injection.

### 2.3. Therapeutic Effect 6 Weeks Post AbobotulinumtoxinA (Dysport^®^) IC

**Figure 2:**
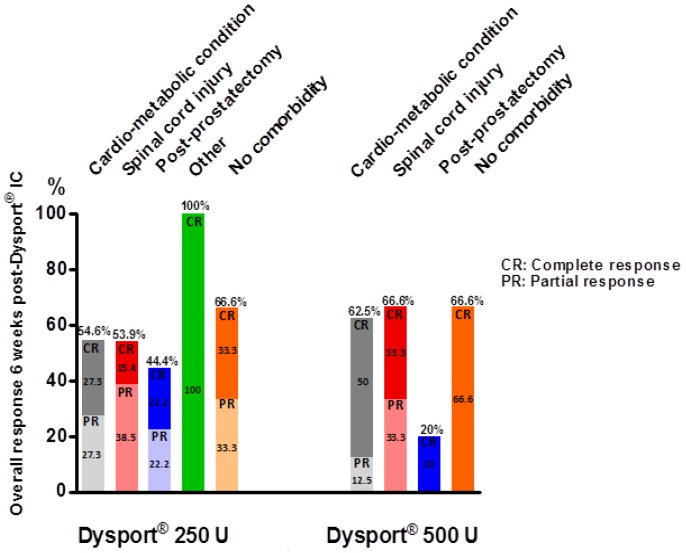
Partial and complete response rates to abobotulinumtoxinA IC (250 and 500 U) combined with PDE5-Is or PGE1 ICI at week 6 post treatment according to ED etiologies and/risk factors.

**Figure 3:**
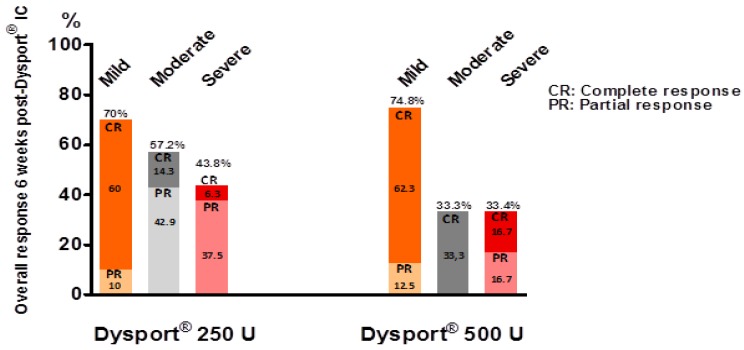
Partial and complete response rates to abobotulinumtoxinA IC (250 and 500 U) at week 6 according to the degree of severity of ED under treatment based on the EF-IIEF domain score [16].

Six ± 2.8 weeks after abobotulinumtoxinA IC, 27 patients were responders (54%) including 15 complete responders (30%) and 12 incomplete responders (24%). Among responders, 23 (85.2%) were still using the same pharmacological ED treatment and 4 (14.8%) have modified the treatment they were using prior to abobotulinumtoxinA IC. Three patients decreased the dose of PGE1 ICI and one treated by a combination of PGE1 ICI with sildenafil stopped sildenafil. At 6 weeks, the average increase in IIEF-EF domain score was 4.7 ± 4.5 (12.3 ± 5.6 prior to Dysport^®^ IC) and 5.6 ± 5.8 (from 14.8 ± 6.6) in the 250 U and 500 U treated groups respectively. Following 250 and 500 U abobotulinumtoxinA IC, the overall response rate at week 6 was respectively 54.5% and 52.9%. The partial and complete response rates to abobotulinumtoxinA IC 250 and 500 U according to i) the different etiologies and/risk factors and ii) severity of ED, are displayed respectively on Figure 2 and Figure 3.

## 3. Discussion

This retrospective case study provides the first report on 6-week post-treatment tolerability and efficacy of intracavernosal injections of abobotulinumtoxinA (Dysport^®^) 250 or 500 U as add on therapy to PDE5-Is or PGE1 IC for ED in patients with insufficient response to those treatments. There was overall a 54% response rate in this difficult-to-treat population, which is quite high. The therapeutic efficacy did not seem to be much influenced by the etiologies and/or risk factors for ED. Conversely, the less severe ED, according to the IIEF-EF domain, scored higher percentages of overall response and complete response. Regarding a possible dose-response effect, the sample size and methodology do not allow to draw any conclusion. Among the responders to abobotulinumtoxinA 250 U, seven were re-evaluated at 21.1 weeks ± 6.3 post treatment (data not shown). Four of them were still responders despite a decrease in the IIEF-EF domain score over time. Conversely, three patients were no longer responders and have requested for a second IC injection of abobotulinumtoxinA. None of them have reported any side-effect. Albeit preliminary, these data tend to indicate that the efficacy of abobotulinumtoxinA 250U could last several months. Such a duration of effect would be in line with the duration of effect of intradetrusor BTX-A for the treatment of neurogenic detrusor activity [17] or BTX-A skin injection for hyperhidrosis, both targeting autonomic innervation [18]. Regarding intradetrusor BTX-A injections, this makes perfect sense that in two different smooth muscle anatomical structures i.e., the detrusor muscle and the corpora cavernosa, the duration of efficacy of BTX-A is comparable. Because abobotulinumtoxinA 500 U IC injections have been performed more recently, preliminary data about the duration of effect and need for re-injection were not available at the time of writing.

Penile erection involves the relaxation of arterial blood supply to the erectile tissue and the trabecular meshwork of smooth muscle that constitutes the paired corpora cavernosa. The main mediator of penile erection is NO [19,20]. NO derived from neuronal NO synthase in the parasympathetic nerves supplying the penis initiates this process. NO is considered as an unconventional neurotransmitter because it is not released by exocytosis. Then, sustained production of NO from endothelial NO synthase within the vascular and trabecular endothelium of the corpora cavernosa released as a paracrine is responsible for full erection and maintenance of erection [21,22]. NO is a freely diffusible gas which has the potential to travel quickly in any direction from its point of production.

It is well known that direct application of clostridium botulinum neurotoxins can elicit long-term disruption of synaptic transmission. The catalytic light chains of these toxins are zinc-dependent endoproteases that cleave conserved SNARE family proteins that are critical for vesicle docking and fusion with the plasma membrane [23,24,25]. Consequently, botulinum neurotoxins cannot impair NO release which is independent of synaptic vesicles. In contrast, there is a permanent sympathetic tone applied to penile vessels and cavernosal smooth muscle fibers responsible for flaccidity that can be blunted by the local delivery of botulinum toxin. Indeed, the sympathetic tone relies on the release of norepinephrine by postganglionic sympathetic neurons occurring by exocytosis from large dense cored synaptic vesicles [26]. A sympatholytic effect of BTX-A has been repeatedly reported in different experimental settings in various animal species [27,28,29]. In a random pattern skin flap model, onabotulinumtoxinA increased vascular blood flow and viable flap area in rats by reducing norepinephrine level while NO was not affected [30]. Furthermore, the antierectile role of the sympathetic innervation has been evidenced in rats [31].

Thus, there is a robust rationale for BTX-A IC to facilitate long-acting cavernosal smooth muscle relaxation through an alteration of the balance within the erectile tissue between the permanent contractile tone applied onto cavernosal smooth muscle cells and the relaxed state of the same cells elicited by the activation of the NO-cGMP pathway. Indeed, such a balance between contractile and relaxant factors governs flaccidity and rigidity. For penile erection to occur, activation of relaxing and inhibition of contractile mechanisms are mandatory [32].

Sympathetic overactivity responsible for an increase in vascular and corporal smooth muscle tone in the erectile tissue is likely involved in the pathophysiology of ED in many patients. Indeed, sympathetic overactivity has been reported to be associated with most of the risk factors and causes of ED including hypertension [33], ischemic heart disease [34], chronic heart failure [35], type 2 diabetes mellitus [36], metabolic syndrome [37], sleep apnea [38], post-radical prostatectomy [39], after spinal cord injury [40], lower urinary tract symptoms [41], and depression [42]. Nevertheless, despite several attempts, it has not been possible to get reliable data about penile sympathetic hyperactivity in ED patients [43].

Overall, we believe that IC BTX-A may improve the efficacy of oral or injectable agents by eliciting i) a decrease in tone of resistant vessels thereby enhancing penile blood flow and ii) an inhibition of the persistent cavernosal smooth muscle tone. The combination of IC BTX-A with pro-erectile drugs is further supported by previous reports of the mild efficacy of a couple of approaches to decrease sympathetic tone within the erectile tissue. A formulation for ICI of moxisylite, a selective 1 blocker, was registered in the 90’s for the treatment of ED [44]. The compound was withdrawn a few years after its launch because of poor efficacy. Cases of prolonged erections were reported following lesions of the paravertebral sympathetic chain at the lumbar level [45], which provides sympathetic innervation to the erectile tissue [46]. It is noteworthy that in the placebo-controlled study with IC onabotulinumtoxinA 50U, with 100 mg of sildenafil on-demand, 7 out of 12 patients in the treatment group could achieve vaginal penetration compared to 2 patients from the control group [12].

This was a retrospective uncontrolled case study with obvious limitations. Based on these preliminary findings, there is need for randomized placebo-controlled trials in order to further investigate the safety and efficacy of abobotulinumtoxinA IC to salvage PDE5-Is and PGE1 IC insufficient responders when combined to existing pharmacological treatments. The response rate from the present pilot study should guide the calculations of the sample size for the next randomized controlled trials.

It is noteworthy that in the study of onabotulinumtoxinA IC [12], the placebo response was very low. This was likely because these patients had already failed oral and injectable treatments. Accordingly, we postulate that in this population of ED patients that are poor responders to existing pharmacological treatments, the fairly high response rate we found when abobotulinumtoxinA was combined to these treatments reflects the therapeutic potential of BTX-A as add-on therapy to PDE5-Is or PGE1 IC for difficult-to-treat patients. Regarding safety, we acknowledge the short-term follow-up of most of the patients in the present report. Nevertheless, despite the paucity of published data [47], according to the registration dossiers of ona, abo and incobotulinumtoxinA for different indications, it appears that the vast majority of the adverse effects caused by BTX-A injections occur in the first weeks post-injection, whatever the site of injection. In the present case studies, patients reported neither adverse events related to BTX-A general diffusion nor local side-effects i.e., ejaculatory disorder, urinary retention, or pelvic floor muscle weakness. In addition, neither prolonged erection nor priapism was observed. This is reassuring regarding the general and locoregional safety of IC BTX-A.

## 4. Conclusions

Although some alternatives exist for patients who are proven non-responders to PDE5-Is, including vacuum constriction devices, vasoactive agents ICI such as PGE1, and ultimately implantation of penile prostheses, failure to achieve successful intercourse after using the maximum recommended dose of PDE5-Is is always a problem if the patient does not desire such alternative treatments. The treatment strategy we propose with IC abobotulinumtoxinA (Dysport^®^) as an add-on therapy might maximize the response rate to PDE5-Is with an acceptable safety. Further clinical research is now mandatory to confirm these preliminary findings.

## 5. Materials and Methods

This study was conducted in a tertiary center specialized in the management of sexual dysfunctions especially in neurologic patients. Based on recent findings of a new indication for BTX-A for the treatment of ED [12], we have treated ED patients with abobotulinumtoxinA IC. A convenience sampling of 47 patients already treated for ED for at least 3 months and with insufficient response to existing pharmacological treatments for ED i.e., PDE5-Is or PGE1 IC, was consecutively recruited in our Neuro-Urology-Andrology unit. Our department has the largest activity in the country with an extensive experience of BTX-A for daily clinical practice and clinical trials for various indications including spasticity, neurogenic detrusor overactivity and idiopathic overactive bladder.

A follow-up visit occurred on average 6 weeks post abobotulinumtoxinA IC. Prior to this follow-up visit, patients were advised to attempt sexual intercourse using their usual pharmacological treatment. Thus, participants completed a minimum of two visits at week 0 at the time of abobotulinumtoxinA IC injections and approximately 6 weeks later. We have retrospectively reviewed medical files from consecutive patients with an insufficient response to pharmacological treatment for ED who have been treated with abobotulinumtoxinA (Dysport^®^) IC as an add-on therapy between January 2018 and March 2019. An analysis of safety and efficacy of abobotulinumtoxinA IC was conducted at the first follow-up visit. The first participant’s first visit and last participant’s last visit occurred in January 2018 and April 2019, respectively.

### 5.1. Eligibility Criteria

Inclusion criteria were: male patients aged >18 years, diagnosed with ED, with a heterosexual stable relationship and being insufficient responders to (i) the maximal registered dose of PDE5-Is: Either 100-mg on-demand sildenafil, 20 mg tadalafil, 20 mg vardenafil, or daily 5 mg tadalafil, or (ii) ICI of PGE1 from 20 to 60 µg. The treatment should have been taken for at least three months. Insufficient response was defined by an Erectile Function domain score of the International Index of Erectile Function (EF domain of the IIEF) <26 on treatment. The International Index of Erectile Function (IIEF) is a 15-item, 5-domain, psychometrically-validated PRO questionnaire [48] that is widely used for the assessment of male sexual function in clinical trials and clinical practice [49,50]. The six-item erectile function (EF) domain of the IIEF is a sensitive and specific measurement of treatment-related changes in EF [48,50]. Exclusion criteria were contraindications to BoNT-A drug monography [51].

### 5.2. Intracavernosal Injections of AbobotulinumtoxinA

Prior to abobotulinumtoxinA IC delivery, an adjustable penile loop ring was placed around the penile shaft by the physician at the crus according to the technique already described [12] while the patient was pulling up his penis. Then, two intracavernosal injections of abobotulinumtoxinA in both corpora cavernosa of 0.5 mL per side were performed using two syringes connected to a 13-mm-long 29 ½ G needle. Then, the penile loop ring was removed after 30 min. The following total doses of abobotulinumtoxinA were delivered: 250 and 500 units Speywood. That means that the concentration for each intracavernosal injection was 250 U or 500 U per mL. The dose of 250 U was tested first, followed by the dose of 500 U to further investigate the safety aspects.

### 5.3. Clinical Endpoints

Side effects were identified by patients’ self-report. Efficacy analysis was based on IIEF-EF improvement. At the first follow-up visit 6 weeks after abobotulinumtoxinA IC, the following definition for responders was implemented: (i) complete responders: IIEF-EF ≥ 26; (ii) partial responders: IIEF-EF < 26 and increase in IIEF-EF versus baseline ≥ 4 and (iii) non responders: IIEF-EF < 26 and increase in IIEF-EF versus baseline < 4 [16]. Any change in pharmacological treatment for ED (PDE5-Is or PGE1 ICI) was captured.

### 5.4. Statistical Analyses

Data were expressed as mean ± SEM. No formal sample size calculation was performed, and all analysis were exploratory. Statistical analysis was performed using Excel v.16 software (Microsoft Office–Redmond, WA, USA, 2016).

### 5.5. Ethics

A written informed consent was obtained from participating patients. According to French legislation for retrospective studies, our database was approved by the French Data Protection Authority (Commission Nationale Informatique et Libertés) under the registration number 2209010 v 0 (22 October 2018). Patients’ medical files were anonymized and patients were informed they can deny access to their personal and medical data at any time.

## Figures and Tables

**Table 1:** Risk factor(s) and etiology(ies) of ED for the 47 patients treated by abobotulinumtoxinA IC (250 and 500 U) insufficient responders to PDE5-Is or PGE1 ICI. “Cardio-metabolic” include diabetes. The total is superior to 100% because for some patients there was more than one risk factor and/or etiology for ED.

ED Risk Factor/Etiology	Cardio-Metabolic	Spinal Cord Injury	Post-Radical Prostatectomy	Other	No Identified Organic Risk Factor/Etiology
*n* (%)	18 (38)	16 (34)	11 (23)	1 (2)	7 (15)
